# Can the dynamic spectral imaging (DSI) color map improve colposcopy examination for precancerous cervical lesions? A prospective evaluation of the DSI color map in a multi-biopsy clinical setting

**DOI:** 10.1186/s12905-020-01169-1

**Published:** 2021-01-12

**Authors:** Berit Bargum Booth, Lone Kjeld Petersen, Jan Blaakaer, Tonje Johansen, Henrik Mertz, Katja Dahl, Pinar Bor

**Affiliations:** 1 Department of Gynecology and Obstetrics, Aarhus University Hospital, Aarhus, Denmark; 2 Department of Gynecology and Obstetrics, Randers Regional Hospital, Skovlyvej 15, 8390 Randers, Denmark; 3 Department of Clinical Medicine, Aarhus University, Aarhus, Denmark; 4 Department of Gynecology and Obstetrics, Odense University Hospital, Odense, Denmark; 5 OPEN Open Patient Data Explorative Network, University of Southern Denmark, Odense, Denmark; 6 Department of Clinical Research, University of Southern Denmark, Odense, Denmark; 7 Department of Pathology, Randers Regional Hospital, Randers, Denmark

**Keywords:** Cervical intraepithelial neoplasia, Colposcopy, Sensitivity, Dynamic spectral imaging

## Abstract

**Background:**

Colposcopy serves as a subjective examination of the cervix with low sensitivity to detect cervical intraepithelial dysplasia (CIN) grade 2 or worse (CIN2 +). Dynamic spectral imaging (DSI) colposcopy has been developed to provide an objective element to cervix examinations and has been proven to increase sensitivity of detecting CIN2 + . We aimed to assess the performance of the DSI color map and compared it to histological diagnoses of cervical biopsies in determining the CIN grade present.

**Methods:**

Women were included in a consecutive, prospective manner at Randers Regional Hospital, Denmark. Women were eligible to participate if they were referred for colposcopy due to abnormal cervical smear (threshold:  ≥ ASCUS) or follow-up after previously diagnosed CIN. All women had four biopsies taken, one directed by colposcopists alone prior to viewing the DSI color map, one directed by the worst color on the respective DSI color map, and two additional biopsies. All biopsies were analyzed separately. We calculated sensitivity, specificity, positive predictive values, and negative predictive values (NPVs) with 95% confidence intervals (CIs).

**Results:**

A total of 800 women were recruited. Of these, 529 (66.1%) were eligible for inclusion. The sensitivity of the DSI color map was found to be 48.1% (95% CI 41.1–55.1) in finding CIN grade 2 or worse (CIN2 +) when compared to the histological diagnosis of the DSI directed biopsy. This was 42.5% (95% CI 36.7–48.5) when compared to the final histological diagnosis of all four cervical biopsies and with an NPV of 53.5% (95% CI 50.5–56.5).

**Conclusion:**

The worst color indicated by the DSI map might not consistently reflect the true grade of cervical dysplasia present. Thus, even though the DSI color map indicates low-grade changes, colposcopists should still consider taking biopsies from the area as high-grade changes might be present.

*Trial registration*: NCT04249856, January 31 2020 (retrospectively registered).

## Background

Colposcopy has been used since the 1920s to examine the uterine cervix by magnifying the transformation zone. Potential signs of precancerous cervical lesions are evaluated and cervical punch biopsies are taken to provide a histological diagnosis of any cervical intraepithelial neoplasia (CIN) or carcinoma. In many countries, however, biopsies are not mandatory part of every colposcopy procedure [1–3]; consequently, it is important for colposcopists to perform accurate evaluations of the cervix. Colposcopy, however, is a subjective examination, the performance of which depends on the colposcopist's experience. The sensitivity to detect CIN grade two or worse (CIN2 +) has been found as low as 55% [4–6].

New digital technologies have been developed to provide a more objective, reproducible evaluation of the cervix and to assist colposcopists in choosing the area(s) to biopsy. One such technology is dynamic spectral imaging (DSI) colposcopy (DySIS Medical Ltd., Edinburgh, UK), which can increase the sensitivity of CIN2 + detection by colposcopy from 55 to 88% [7]. The DSI colposcope measures the duration and density of any aceto-white changes that may occur on the cervix and, based on these registrations, provides a color-coded map to guide colposcopists during biopsy. The colors provided by the DSI color map can indicate the severity of precancerous cervical lesions [7–16]. Other epithelial changes on the cervix, such as punctuations and mosaic patterns, are also usually considered during colposcopy as potential indicators of CIN, but these changes are not part of the DSI evaluation.

It is essential that the DSI color map directs colposcopists to the most worrisome area(s) to improve the detection of CIN and prevent precancerous cervical lesions from being missed in women. The objective of this study is therefore twofold: (1) to compare the worst color on the DSI color map to the histological diagnosis of the biopsy taken from the worst indicated area and (2) to compare the worst color on DSI color map to the final histological diagnosis (FHD) found by four cervical punch biopsies. These comparisons determine whether the DSI color map can correctly guide the colposcopists in taking biopsies from the most severe intraepithelial lesions.

## Methods

Women were included in a prospective, consecutive, non-randomized manner at the outpatient clinic of Randers Regional Hospital between 1 February 2017 and 31 December 2019. All colposcopy referrals were considered, including women screened by invitation in the national screening program and women examined with symptoms. Women were eligible to participate if they were over 18 years of age and were referred to colposcopy with an abnormal cervical smear that is, atypical squamous cells of undetermined significance (ASCUS), low-grade squamous intraepithelial lesions (LSILs), atypical squamous cells favoring high grade (ASC-H), high-grade squamous intraepithelial lesions (HSILs), atypical glandular cells (AGCs), adenocarcinoma in situ (AIS), adenocarcinoma (ACC), or squamous cell carcinoma (SCC). In women above the age of 29 and an ASCUS cytology sample, HPV testing was routinely performed. Women who underwent a follow-up colposcopy due to a previous CIN diagnosis were also included. Women who had cervical punch biopsies less than six months prior were excluded. Other exclusion criteria were previous cervical conization, current pregnancy or pregnancy in the prior three months, and previous pelvic radiation therapy. Women with a non-visible transformation zone and those with one or more cervical biopsies unsuitable for pathological analysis were excluded from the analysis. Women eligible to participate were identified by colposcopists who informed them about the study. The participants gave oral and written consent and provided information on their height, weight, smoking habits, previous pregnancies, contraception use, and human papillomavirus (HPV) vaccination status in a short baseline data sheet.

All women were examined by DSI (DySIS, V3) colposcope and all colposcopists were instructed in how to use the DSI colposcope. As recommended by the Danish national guidelines, each woman had four cervical punch biopsies taken at the end of the colposcopy procedure [17]. The DSI color map is based on a range of colors from cyan to blue to green to red to yellow to white, which represent increasing severity. The worst color shown on the map was recorded by the colposcopists. Based on previous publications and the *DySIS Medical Instructions for Use*, the colors cyan, blue, and green were considered to represent low-grade cervical changes, whereas red, yellow, and white were considered to represent high-grade cervical changes (CIN2 +) [8, 10, 12, 14–16, 18].

After the automatic application of acetic acid, the DSI colposcope initiated analysis of the cervix. During this time (125 s), the colposcope was used as a conventional colposcope, and the colposcopist performed his/her own analysis of the cervix. Before the DSI color map was revealed, the colposcopist marked on the build-in screen the first biopsy site, that is, the area the colposcopist viewed as the most abnormal [biopsy number one, the colposcopy-directed biopsy (CDB)]. Biopsy number two was marked according to the area the DSI color map indicated as the worst. Biopsy numbers three and four were then marked as additional biopsies and taken from two quadrants not represented in the first two biopsies. If the colposcopist and DSI color map agreed on the worst area, one biopsy was taken from that area and then three additional biopsies. All biopsy records were stored in the DSI system. Each biopsy was taken with 3-mm forceps and placed in a separate container with the corresponding number and sent for analysis, which was made by one of two gynecological pathologists in the Pathology Department of the Regional Hospital in Randers. Pathologists were blinded to number and origin of the biopsy and each biopsy diagnosis was recorded separately. The final histological diagnosis (FHD) was based on the worst of the four cervical punch biopsies, and the participants were subsequently treated according to the Danish national guidelines [17] i.e. see-and-treat was not used in our setting. Low-grade lesions were treated conservatively as well as CIN2 (and ungradable CIN) in women who had further fertility desires or preferred not to undergo Loop Electrosurgical Excision Procedure (LEEP). CIN3 + was treated by LEEP.

Colposcopists recorded their own clinical backgrounds (trained colposcopy nurses, resident doctors, or consultant doctors), type of transformation zone seen (fully visible, partially visible, or not visible), visible signs of dysplasia, and their own colposcopic impressions.

We defined low-grade referrals as women referred due to ASCUS or LSIL cytology or follow-up colposcopy due to previous CIN grade 1 (CIN1) diagnoses. High-grade referrals were defined as women referred to colposcopy due to ASC-H, HSIL, AIS, AGC, ACC, SCC, or for a follow-up colposcopy due to previous CIN2 + diagnoses. We also chose to classify ungradable CIN with CIN2 in our analysis, as these women were most likely to have follow-ups in six month intervals, which was the same as the recommended follow-up time for CIN2 in the national guidelines.

### Statistical analysis

Sensitivity, specificity, positive predictive values (PPVs), and negative predictive values (NPVs) were calculated with 95% confidence intervals (CIs). We assumed the FHD based on the four cervical punch biopsies to represent the true dysplasia grade. Our previous study showed a concordance of 95% between the FHD and the conization specimen [19]. Stata 16.1 analytic software (Stata Corp LP, College Station, TX) was used for the statistical analyses.

### Ethics

The Central Denmark Region Committees on Biomedical Research Ethics determined that the project was exempt from approval; it was viewed as a quality-improvement study (jr.nr. 1-10-72-262-16). The study was approved by the Danish Data Protection Agency (jr.nr. 1-16-02-534-16).

## Results

**Fig. 1:**
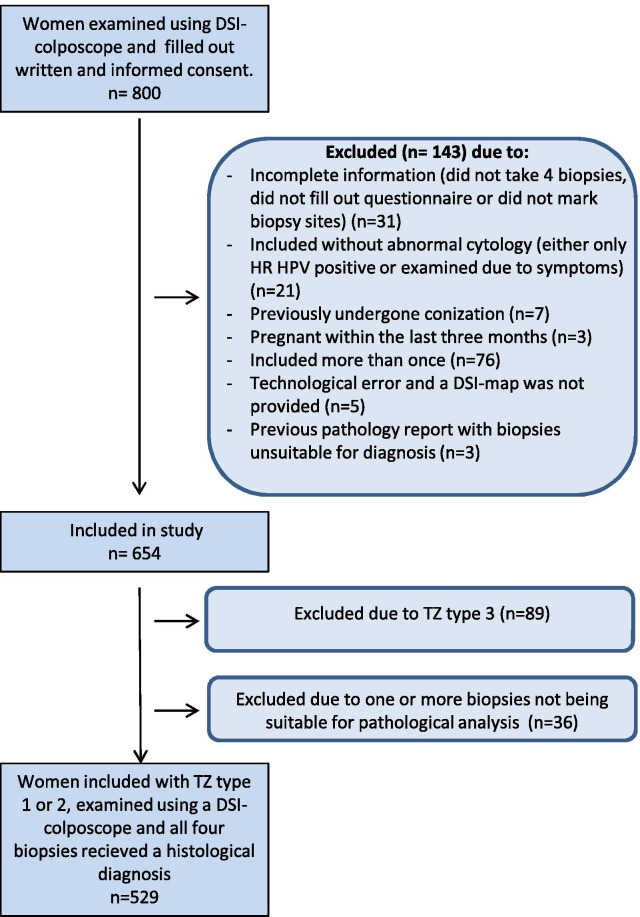
Participant flow diagram. *DSI* dynamic spectral imaging, *TZ* transformation zone, *TZ type 1* fully visible, *TZ type 2* partially visible, *TZ type 3* not visible

A total of 800 women were examined by DSI colposcopy at our department during the study period. Of these, 529 (66.1%) fulfilled all the study inclusion criteria (see Fig. 1).

Women included in the study had a median age of 30.3 years (range 19.7–72.8) (Table 1). The majority, 87.7% (n = 464), were new referrals due to an abnormal smear, of which just over half (n = 263, 56.7%) were referred with low-grade cytology and 43.3% (n = 201) with high-grade cytology. Despite being eligible for inclusion, no women were referred with invasive cytology during the study period. The remaining 12.3% (n = 65) were seen for follow-up of previously diagnosed CIN. Colposcopy procedures were most frequently undertaken by trained nurse colposcopists (70.7%), followed by resident doctors (17.2%) and consultant doctors (12.1%). HPV testing was performed in 122 women (23.1%). Colposcopists reported aceto-white changes in 87.0% of women. See Table 1 for further baseline characteristics of the women included in the study (Table 2).

**Table 1:** Baseline demographic and clinical characteristics of participants

	Number of women N (%)
Total	529
Age median (range)	30.3 (19.7–72.8)
BMI median (range)^a^	23.1 (16.8–59.5)
Smoking	
No	269 (50.9)
Current	128 (24.2)
Previous	132 (24.9)
Contraception use	
Oral	204 (38.6)
IUD	102 (19.3)
Condom	26 (4.9)
Other	8 (1.5)
None	189 (35.7)
Parity	
No previous pregnancies	226 (42.7)
Previous births	173 (32.7)
Previous abortions (spontaneous and provoked)	36 (6.8)
Both	94 (17.8)
HPV vaccination status	
Not vaccinated	190 (35.9)
Vaccinated	318 (60.1)
Ongoing	14 (2.7)
Unknown	7 (1.3)
HPV tested	
No	407 (76.9)
Yes	122 (23.1)
HPV status	122
HPV 16	12 (9.8)
HPV 18	5 (4.1)
Other hrHPV	93 (76.2)
HPV 16 + other hrHPV	7 (5.8)
HPV 18 + other hrHPV	1 (0.8)
No HPV found	4 (3.3)
New referral	464
ASCUS	167 (36.0)
LSIL	96 (20.7)
ASC-H	55 (11.8)
AGC	4 (0.9)
HSIL	142 (30.6)
Follow-up	65
CIN1	10 (15.4)
CIN2	41 (63.1)
CIN3	3 (4.6)
Ungradable CIN	11 (16.9)
Colposcopists	
Nurse colposcopist	374 (70.7)
Resident	91 (17.2)
Consultant	64 (12.1)
TZ visible?	
Yes, fully	378 (71.5)
Yes, partially	151 (28.5)
What lesions were visible?	
Aceto-whitening	460 (87.0)
Atypical vessels	59 (11.2)
Punctuations	90 (17.0)
Mosaic pattern	100 (18.9)
No visible lesions	47 (8.9)

*BMI* body mass index, *IUD* intra-uterine device, *HPV* human papilloma virus, *hrHPV* high-risk HPV, *ASCUS* atypical squamous cells of undetermined significance, *LSIL* low-grade squamous intraepithelial lesion, *ASC-H* atypical squamous cells favoring high grade, *HSIL* high-grade squamous intraepithelial lesion, *AGC* atypical glandular cells, *TZ* transformation zone

^a^5 women missing BMI information

**Table 2:** Comparisons of DSI-directed biopsy histological diagnosis and the worst color indicated by the DSI color map

Worst color indicated by DSI color map	Histological diagnosis of the DSI-directed biopsy		Total
	≤ CIN1	CIN2 +^a^	
Low-grade (cyan, blue, green)	247	108	355
High-grade (red, yellow, white)	74	100	174
Total	321	208	529

^a^Ungradable CIN was included in the CIN2 category, as they were most likely to be referred for follow-up

In 64.1% of the colposcopy procedures, the colposcopists and the DSI color map chose the same area, and in 35.9% of the women, the colposcopists and the DSI color map chose different areas.

**Fig. 2:**
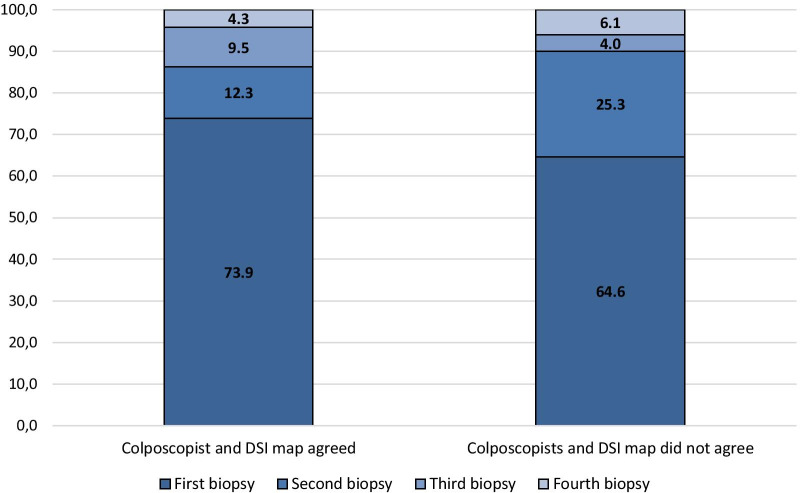
Additional percentage of CIN2 + diagnoses found per additional biopsy

In total, 287 women (54.3%) had an FHD of CIN2 + . For low-grade referrals, this was 37.3%, and for high-grade referrals, it was 72.3%. Conization by LEEP was performed in 186 women (35.2%). We found a concordance of 95.7% between the histological diagnosis of the cone specimen and the FHD based on the four cervical punch biopsies in this subgroup. Women found with CIN2 who did not undergo conization were managed conservatively and followed after six months, in accordance with national guidelines. In the 287 women who had a FHD of CIN2 + we calculated the percentage yield of CIN2 + for each additional biopsy based on whether the colposcopist and the DSI map agreed on the most abnormal area, see Fig. 2. When the DSI map and the colposcopists agreed on the area to biopsy, 73.9% of the CIN2 + diagnoses were found in the first biopsy. When they did not agree, 64.4% of CIN2 + diagnoses were found in the CDB and this was increased to 89.9% when adding the DSI-directed biopsy. There was no significant difference between the percentage of CIN2 + found between the groups when two biopsies were considered in both groups (86.2% vs. 89.9%, p = 0.4).

Colposcopists recorded the worst color observed on the DSI color map as 28.2% (n = 149) cyan, 23.2% (n = 123) blue, 15.7% (n = 83) green, 13.4% (n = 71) red, 7.4% (n = 39) yellow, and 12.1% (n = 64) white, meaning that 67.1% (n = 355) of the women were indicated to have low-grade cervical dysplasia and 32.9% (n = 174) to have high-grade cervical dysplasia by the DSI color map.

### Dynamic spectral imaging color map versus the DSI-directed biopsy

**Fig. 3:**
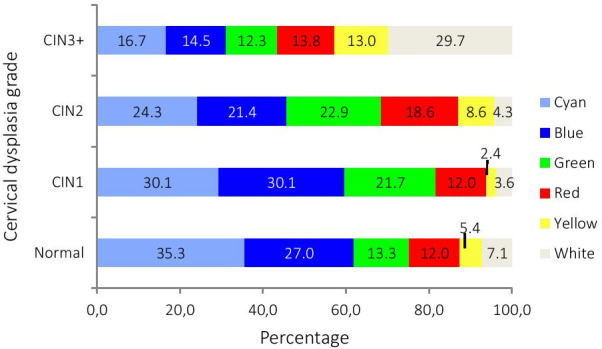
Percentage of worst colors on the DSI color map compared to dysplasia grades found in DSI-directed cervical punch biopsies

We compared the worst color indicated by the DSI color map to the histological diagnosis of the DSI-directed biopsy, the distribution between which can be seen in Table 3 (and Additional file 1). A trend of increased high-grade coloring by the DSI color map with an increasing severity of dysplasia diagnosis was found when the worst color indicated by the DSI map was compared to the histological diagnosis of the DSI-directed cervical biopsy (Fig. 3).

**Table 3:** Comparison of the final histological diagnosis and the worst color on the DSI color map

Worst color indicated by DSI color map	Final histological diagnosis of the four cervical punch biopsies		Total
	≤ CIN1	CIN2 +^a^	
Low-grade (cyan, blue, green)	190	165	355
High-grade (red, yellow, white)	52	122	174
Total	242	287	529

^a^Ungradable CIN was included in the CIN2 category, as they were most likely to be referred for follow-up

The DSI color map had a sensitivity of 48.1% (95% CI 41.1–55.1) and a specificity of 77.0% (95% CI 72.0–81.4) to correctly identify CIN2 + when compared to the DSI-directed biopsy. For low-grade referrals, sensitivity was 44.1% (95% CI 32.1–56.7), and for high-grade referrals, sensitivity was 50.0% (95% CI 41.4–58.6). The PPV of the DSI color map compared to the DSI-directed biopsy alone was 57.5% (95% CI 51.4–63.2), and the NPV was 69.6% (95% CI 66.5–72.5) (Table 4).

**Table 4:** Performance of dynamic spectral imaging color map in identifying high-grade cervical dysplasia (CIN2 +)

Final histological outcome from four cervical punch biopsies	DSI color code compared to DSI-directed biopsy	DSI color code compared to final histological diagnosis
All referrals		
Sensitivity	48.1% (95% CI 41.1–55.1)	42.5% (95% CI 36.7–48.5)
Specificity	77.0% (95% CI 72.0–81.4)	78.5% (95% CI 72.8–83.5)
PPV	57.5% (95% CI 51.4–63.3)	70.1% (95% CI 64.0–75.6)
NPV	69.6% (95% CI 66.56–72.5)	53.5% (95% CI 50.5–56.5)
LG referrals		
Sensitivity	44.1% (95% CI 32.1–56.7)	37.3% (95% CI 27.9–47.4)
Specificity	79.0% (95% CI 72.8–84.4)	79.5% (95% CI 72.7–85.3)
PPV	41.1% (95% CI 32.4–50.4)	52.1% (95% CI 42.4–61.6)
NPV	81.0% (95% CI 77.3–84.2)	68.0% (95% CI 64.2–71.5)
HG referrals		
Sensitivity	50.0% (95% CI 41.4–58.6)	45.4% (95% CI 38.1–52.9)
Specificity	73.3% (95% CI 64.3–81.1)	76.1% (95% CI 64.5–85.4)
PPV	69.3% (95% CI 61.6–76.1)	83.2% (95% CI 76.0–88.5)
NPV	54.8% (95% CI 49.9–59.7)	34.8% (95% CI 30.8–39.2)

*DSI* dynamic spectral imaging, *PPV* positive predictive value, *NPV* negative predictive value, *LG* low-grade, *HG* high-grade

### Dynamic spectral imaging map versus FHD of four biopsies

Likewise, comparing the worst color observed on the DSI color map to the FHD based on all four biopsies, the DSI color map had a sensitivity of 42.5% (95% CI 36.7–48.5) and a specificity of 78.5% (95% CI 72.8–83.5) to correctly identify CIN2 + when compared to the FHD. The PPV was 70.1% (95% CI 64.0–75.6), and the NPV was 53.5% (95% CI 50.5–56.5). For low-grade referrals, sensitivity was 37.3% (95% CI 27.9–47.4), and for high-grade referrals, it was 45.4% (95% CI 38.1–52.9), with specificity being 79.5% (95% CI 72.7–85.3) for low-grade referrals and 76.1% (95% CI 64.5–85.4) for high-grade referrals (see Tables 3, 4, and Additional file 2 for the distribution between DSI colors and FHD).

**Fig. 4:**
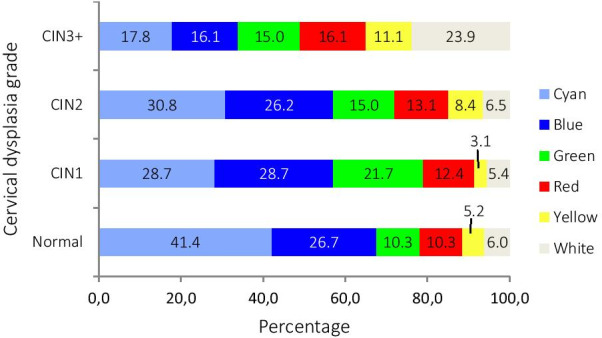
Percentage of worst color on the DSI color map compared to dysplasia grades found based on all four cervical punch biopsies

Figure 4 shows the worst color indicated by the DSI color map according to the histological diagnosis found in all four cervical biopsies. Again, as in Fig. 3, a trend can be seen of increasing high-grade colors shown by the DSI-map as the dysplasia grade increases. However, the lowest-grade-indicating colors blue and green were evenly distributed across the different grades of dysplasia diagnoses in both figures.

In new low-grade referrals (263 women), 99 (37.6%) had an FHD of CIN2 + . In these women who were referred with low-grade cytology but had a high-grade FHD (99 women), the DSI color map showed low-grade colors as the worst in 62 of them (62.6%). More specifically, cyan was the worst color in 28 (28.3%), blue in 19 (19.2%), and green in 15 (15.2%). Of these 99 women, 38 had CIN3 + , and 52.6% of these CIN3 + diagnoses (20 of 38) had colors indicating low-grade disease as the worst color on the DSI color map.

### Histological diagnosis of DSI-directed biopsy compared to FHD of four biopsies

We further calculated performance variables based on the histological diagnosis of the DSI-directed biopsy compared to the FHD of all four biopsies. The DSI-directed biopsy had a sensitivity for CIN2 + detection of 72.5% (95% CI 66.9–77.6); this was 66.7% (95% CI 56.6–75.7) for low-grade referrals and 76.9% (95% CI 70.1–82.8) for high-grade referrals. The NPV was 75.4% (95% CI 71.7–78.7) for all, 83.4% (95% CI 79.3–86.9) for low-grade referrals, and 62.8% (95% CI 56.5–68.8) for high-grade referrals.

### Cervical cancer cases

In total, seven women (1.3%) were diagnosed with cervical cancer following the colposcopy examination. In these women, the worst colors indicated by the DSI color maps were as follows; two cyan (one SCC stage IA1, one adenocarcinoma stage IB1), one blue (SCC stage IB), one green (SCC stage IA1), one red (one SCC stage IA1), one yellow (SCC stage 2B), and one white (SCC stage IA1). Cervical cancer stages were classified according to the The International Federation of Gynecology and Obstetrics (FIGO) 2009 classification. In all the seven cancer cases high-grade histological disease was identified by both the CDB and the DSI directed biopsies.

## Discussion

We found that the DSI color map yielded a sensitivity of 42.5% to correctly identify high-grade dysplasia (CIN2 +) irrespective of the referral diagnosis, compared to the FHD based on four biopsies as the golden standard. This was 37.3% for low-grade referrals and 45.4% for high-grade referrals. Furthermore, comparing the histological diagnosis of the DSI-directed biopsy to the FHD we found the sensitivity to be 72.5%. The NPV was only 53.5% compared to the FHD and was especially low in high-grade referrals: 34.8%.

Louwers et al. [7] found the DSI color map to have a sensitivity to detect CIN2 + of 65% in their intention-to-treat cohort and reported an NPV of 71%. When stratified by referral cytology, they found a sensitivity to detect CIN2 + of 70% and a NPV of 82% in low-grade referrals and a sensitivity to detect CIN2 + of 62% and an NPV of 42% in high-grade [13]. The differences in results were likely due to differences in the respective study designs; Louwers et al. took at least one biopsy in every woman, but all the biopsies taken were analyzed together, so it was not possible to distinguish the histological diagnoses of the biopsies taken from areas indicated by the colposcopists, the DSI color map, and additional biopsies. It was also unclear what DSI color warranted a biopsy to be taken. Finally, as shown by our own data, a major difference can exist between the sensitivity found when comparing the color indicated by the DSI color map to the FHD (42.5%) and the sensitivity found when comparing the histological diagnosis of the DSI-directed biopsy to the FHD (72.5%). In 63.9% of women the colposcopists and DSI color map agreed on the area to biopsy, meaning for these cases the sensitivities found also apply to the colposcopists.

Aceto-white epithelium can represent a wide range of changes on the cervix and is not always representative of precancerous lesions. [20] DSI technology only measures aceto-white changes, and the cervical dysplasia recorded in other biopsies could have been represented by other visual changes. Additionally, lesions may also be invisible from the surface if they are hidden in cervical crypts. Aceto-whitening was observed by the colposcopists in 87.0% of the women in this study. However, the DSI color map only reported colors worse than or equal to blue in 71.8% of women or equal to green or worse in 48.6% of women, which may indicate either that the colposcopists over-reported aceto-white changes and incorrectly interpreted normal epithelial reactions, such as interpreting metaplasia as abnormal, or the DSI color maps underestimated the aceto-white changes. This could be due to the DSI colposcope also measuring fading times of aceto-white changes. Overall, the sensitivities and PPVs were higher for high-grade referrals than low-grade referrals in our study, which may indicate that low-grade lesions are more difficult to identify by the DSI color map. Furthermore, 60.1% of our population was immunized against HPV, and an additional 2.7% had started the immunization process. Zaal et al. [15] reported that the DSI color map could find cervical lesions that involved HPV type 16 better than HPV type 16 negative lesions. With such a high proportion of women immunized in our population, this may also have played a role in the performance of the DSI color map. The majority of HPV types identified in our population were other high-risk types than HPV type 16 and 18. In the future, we expect more women to have received the HPV vaccine, as the 9-valent vaccine is currently offered to both boys and girls at the age of 12 as part of the Danish Childhood Vaccination Program [21]. Additionally, with national screening programs switching to primary HPV testing rather than cytology, we expect women referred for colposcopy to present with smaller lesions than before.

Colposcopy guidelines from the UK and USA state that women with normal colposcopic impressions who are referred with low-grade cytology can be observed without biopsies [1, 2]. In our study, women newly referred with low-grade cytology were found to have high-grade disease (CIN2 +) in 37.6% (99 of 263) of cases. The DSI color map indicated low-grade disease in 62.6% (62 of 99) of these women. Cervical cancers were diagnosed in women who had presented with all the different colors of the DSI color map as the worst color. It is therefore important to emphasize that DSI technology is intended to supplement regular colposcopy examinations, not to be the only factor deciding whether biopsies should be taken.

To the best of our knowledge, no other country but Denmark recommends four biopsies to be taken in all women, and neither has any other study published on DSI technology included four biopsies. In Wales, Budithi et al. [8] reported that only 58% of the women in the study had biopsies taken, while in the USA, Cholkeri-Singh et al. [9] reported that 71.5% of women had biopsies taken during colposcopy, and DeNardis et al. [11] found a similar 75.7% rate. In both these studies from the USA, women had an average of 1.2 biopsies taken, and biopsies were not analyzed separately [8, 9]. Interestingly, these studies reported a much lower rate of CIN2 + in their study cohorts than our data. Budithi et al. [8] found CIN2 + in 16.5% of women in Wales, and even the two studies from the USA reported lower rates of 9.5% and 12.8%, respectively [9, 11]. While we found CIN2 + in 54.3% of women (regardless of referral cytology), Louwers et al. [7, 13], who took at least one biopsy in every woman, found a CIN2 + detection rate of 47%. This further confirms that more biopsies increase the likelihood of finding cervical dysplasia [22]. In countries with a conservative CIN2-management strategy, this will raise the number of women who need follow-ups, especially for lesions that might regress on their own. It is important, however, to know and follow the true dysplasia grade present at colposcopy to offer conservative management on an informed basis. Further analysis on whether a woman presenting with a DSI color map indicating low-grade disease but with a CIN2 diagnosis based on cervical biopsies is more likely to regress spontaneously, while outside the scope of this project, could be of interest for future studies.

As mentioned earlier, the main strength of this study was that all women had four biopsies performed, so we could limit verification bias and approach the true dysplasia grade as close as possible without performing unnecessary conizations. With our study design, we provided the histological diagnosis from each biopsy taken and correctly indicated the degree of cervical dysplasia from the specific area indicated by the DSI color map. The pathologists analyzing the biopsies did not know which biopsy represented which area. However, one limitation of this design is that a single biopsy indicating high-grade disease would influence the FHD, meaning it was not possible to calculate specificity when comparing the histological diagnosis of the DSI-directed biopsy to the FHD, as no biopsy would be considered a false positive, which further limits the statistical comparison of sensitivities, as the different gold standards are not independent of each other. Intra- and interobserver variability in the pathological grading of CIN is another limitation of the study [23]. In addition, the interpretation of the worst color reported by the DSI color map was up to the individual colposcopists, a potential bias because it depends on the way the map was interpreted; nonetheless, this represents the use of the DSI color map in a real-life clinical setting.

It is important to correctly diagnose the degree of cervical dysplasia to be able to offer women conservative management safely. This might allow longer time periods between follow-ups in the future; however, further research is still needed to determine this.

## Conclusion

We found that the worst color indicated by the DSI color map might not be correct in reflecting the worst grade of cervical dysplasia present in women referred with abnormal cervical cytology. Thus, our results show colposcopists should be cautious to avoid misinterpreting colors indicating low-grade dysplasia without biopsy, as there could still be high-grade disease present. Multiple biopsies are recommended.

## Supplementary information

**Additional file 1:** Detailed distribution of worst DSI colors and histological diagnoses of the DSI-directed biopsies.

**Additional file 2:** Detailed distribution of worst DSI colors and final histological diagnosis of all four cervical biopsies.

## Data Availability

Restrictions apply to the availability of these data, which were used under the license of this study. Data are available from the authors upon reasonable request and with the permission from the Danish Data Protection Agency.

## Abbreviations

CDB: Colposcopy-directed biopsy
CIN: Cervical intraepithelial neoplasia
DSI: Dynamic spectral imaging
FHD: Final histological diagnosis
NPV: Negative predictive value
PPV: Positive predictive value
TZ: Transformation zone
