# Supplementary material for: Can the dynamic spectral imaging (DSI) color map improve colposcopy examination for precancerous cervical lesions? A prospective evaluation of the DSI color map in a multi-biopsy clinical setting
Source: BMC Womens Health. 2021 Jan 12;21:21. doi: 10.1186/s12905-020-01169-1 (PMC7802273; doi:10.1186/s12905-020-01169-1)
Supplement: Supplementary file 1 — Additional file 1: Detailed distribution of worst DSI colors and histological diagnoses of the DSI-directed biopsies. [file 12905_2020_1169_MOESM1_ESM.docx]

**Additional file 1: Detailed distribution of worst DSI colors and histological diagnoses of the DSI-directed biopsies**

|  | | **Histological diagnosis of the DSI-directed biopsy** | | | |
| --- | --- | --- | --- | --- | --- |
| **Worst color indicated by DSI color map** | | **Normal** | **CIN1** | **CIN2*** | **CIN3+** |
| **Low-grade** | **Cyan** | 85 | 24 | 17 | 23 |
|  | **Blue** | 63 | 25 | 15 | 20 |
|  | **Green** | 32 | 18 | 16 | 17 |
| **High-grade** | **Red** | 29 | 10 | 13 | 19 |
|  | **Yellow** | 13 | 2 | 6 | 18 |
|  | **White** | 17 | 3 | 3 | 41 |
|  | **Total** | **239** | **82** | **70** | **138** |

*Ungradable CIN was included in the CIN2 category, as they were most likely to be referred for follow-up.
