# Supplementary material for: Can the dynamic spectral imaging (DSI) color map improve colposcopy examination for precancerous cervical lesions? A prospective evaluation of the DSI color map in a multi-biopsy clinical setting
Source: BMC Womens Health. 2021 Jan 12;21:21. doi: 10.1186/s12905-020-01169-1 (PMC7802273; doi:10.1186/s12905-020-01169-1)
Supplement: Supplementary file 2 — Additional file 2: Detailed distribution of worst DSI colors and final histological diagnosis of all four cervical biopsies. [file 12905_2020_1169_MOESM2_ESM.docx]

**Additional file 2: Detailed distribution of worst DSI colors and final histological diagnosis of all four cervical biopsies**

|  | | **Final histological diagnosis of the cervical punch biopsies** | | | |
| --- | --- | --- | --- | --- | --- |
| **Worst color indicated by DSI color map** | | **Normal** | **CIN1** | **CIN2*** | **CIN3+** |
| **Low-grade** | **Cyan** | 48 | 36 | 33 | 32 |
|  | **Blue** | 29 | 37 | 28 | 29 |
|  | **Green** | 12 | 28 | 16 | 27 |
| **High-grade** | **Red** | 12 | 16 | 14 | 29 |
|  | **Yellow** | 6 | 4 | 9 | 20 |
|  | **White** | 7 | 7 | 7 | 43 |
|  | **Total** | **114** | **128** | **107** | **180** |

*Ungradable CIN was included in the CIN2 category, as they were most likely to be referred for follow-up.
